# Radiation-Induced Cardiovascular Disease: Mechanisms and Importance of Linear Energy Transfer

**DOI:** 10.3389/fcvm.2018.00005

**Published:** 2018-01-31

**Authors:** Christopher B. Sylvester, Jun-ichi Abe, Zarana S. Patel, K. Jane Grande-Allen

**Affiliations:** ^1^Department of Bioengineering, Rice University, Houston, TX, United States; ^2^Medical Scientist Training Program, Baylor College of Medicine, Houston, TX, United States; ^3^Department of Cardiology, Division of Internal Medicine, The University of Texas MD Anderson Cancer Center, Houston, TX, United States; ^4^Science and Space Operations, KBRwyle, Houston, TX, United States

**Keywords:** cardiovascular disease, radiation, cancer, charged particle, linear energy transfer, chronic inflammation, space radiation

## Abstract

Radiation therapy (RT) in the form of photons and protons is a well-established treatment for cancer. More recently, heavy charged particles have been used to treat radioresistant and high-risk cancers. Radiation treatment is known to cause cardiovascular disease (CVD) which can occur acutely during treatment or years afterward in the form of accelerated atherosclerosis. Radiation-induced cardiovascular disease (RICVD) can be a limiting factor in treatment as well as a cause of morbidity and mortality in successfully treated patients. Inflammation plays a key role in both acute and chronic RICVD, but the underling pathophysiology is complex, involving DNA damage, reactive oxygen species, and chronic inflammation. While understanding of the molecular mechanisms of RICVD has increased, the growing number of patients receiving RT warrants further research to identify individuals at risk, plans for prevention, and targets for the treatment of RICVD. Research on RICVD is also relevant to the National Aeronautics and Space Administration (NASA) due to the prevalent space radiation environment encountered by astronauts. NASA’s current research on RICVD can both contribute to and benefit from concurrent work with cell and animal studies informing radiotoxicities resulting from cancer therapy. This review summarizes the types of radiation currently in clinical use, models of RICVD, current knowledge of the mechanisms by which they cause CVD, and how this knowledge might apply to those exposed to various types of radiation.

## Introduction

Radiation therapy (RT) has been used since the 1890s to treat cancer. RT can be used as a primary treatment or adjuvant to a combination of surgery, chemotherapy, targeted small molecules, or biologic drugs. Traditionally, low-linear energy transfer (LET) radiation such as photons (X-rays and γ-rays), have been the mainstay of RT, but since the 1950s, charged particle therapy (CPT) in the form of proton beams have been available and have showed superiority to photon therapies against some cancers (1). More recently, therapies using high-LET (densely ionizing) heavy charged particles such as carbon are being used because they can more precisely deliver higher intensity energy while decreasing the dose to healthy tissues in the path of radiation.

Use of high-LET therapies remain limited to small cohorts and most high-LET treatment centers are outside of the United States, primarily in Germany and Japan, but centers are now being built at the University of Texas Southwestern and University of California San Francisco medical centers in the United States (2). The complications of low-LET RT exposure have been well-reviewed both in this journal (3) and elsewhere (4–6), but the effects of high-LET radiation from heavy charged ions are not well-characterized. Aside from its use as RT, the effects of high-LET radiation are relevant to the National Aeronautics and Space Administration (NASA) because of possible effects of space radiation on astronauts during extended missions.

Radiation-induced cardiovascular disease (RICVD) is one well-known complication of low-LET radiation exposure. RICVD can occur in individuals at otherwise low risk for cardiovascular disease (CVD) (7, 8), or it can exacerbate existing CVD (9, 10). The incidence of most cancers increases with age, as does the prevalence of traditional CVD. Thus, the population that most often needs treatment with radiation is the most at risk for complications of RICVD. RICVD can be a treatment-limiting factor in those who receive RT, especially to the thorax (9–11). Evidence suggesting that radiation independently causes CVD includes its development after radiation exposure in healthy or younger populations in whom the disease is almost uniformly absent (8, 12); the development of RICVD in areas directly exposed to radiation (7, 13); and accelerated progression of chronic CVD in at-risk or affected individuals (10). In addition to studies of patients exposed to therapeutic radiation, several groups exposed to nuclear radiation occupationally have had longitudinal follow-up (6). Study of these therapeutically and occupationally exposed groups has revealed a temporally bimodal distribution of RICVD. Short-term effects of RICVD such as acute pericarditis occur within weeks after doses >30 Gy (4). Long-term effects of RT such as atherosclerosis and coronary artery disease manifest more than a decade after exposure (4). High-LET radiation may affect the cardiovascular system in a different manner than that of traditional low-LET radiation (14, 15), and study of how high-LET radiation affects the cardiovascular system is underway. This review will focus on the available data on the effects of low- and high-LET radiation on the cardiovascular system, and how these results may impact those who will be exposed to high-LET radiation in the future.

## Comparison of Low-Let and High-Let Modalities

Linear energy transfer refers to the amount of energy deposited into a material as an ionizing particle passes through it. Energy deposition, and thus ionization, of a beam increases with increasing LET. The exact LET depends on both the radiation type and the material traversed. Because of the heterogeneity of biological materials, LET must be considered in the context of both the tissue being irradiated and the type of radiation being transmitted, but in general LET increases with the mass of the particles used for irradiation.

**Figure 1:**
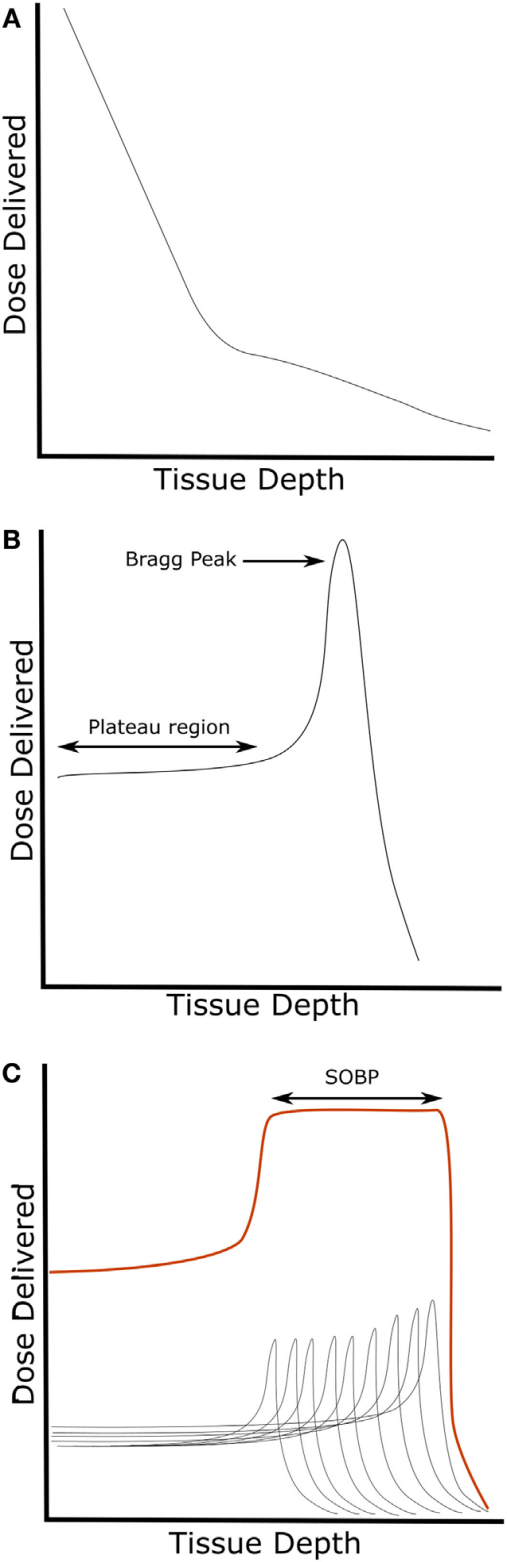
Representative dose delivery patterns of **(A)** low-LET photons and **(B)** charged particles. **(B)** Charged particles demonstrate a low-LET plateau region before the high-LET Bragg peak. **(C)** Exposures with varying initial energies (gray lines) can be used to create a SOBP (red line) and cover the entire volume of targeted tissue with approximately the same dose of radiation. Abbreviations: LET, linear energy transfer; SOBP, spread-out Bragg peak.

External beam low-LET photon RT (X-rays or γ-rays) has been the primary modality of RT since its first clinical implementation and has remained the most commonly used modality of RT. Photons typically deliver the greatest dose of radiation at the first surface of tissue encountered, and the dose delivered decreases linearly as the beam pass through tissues (Figure 1A). The linear dose delivery of photon therapies means that healthy tissues in the path of the beam may also be damaged. Two techniques, dose fractionation and conformal radiation, have been used to reduce the amount of damage to off-target tissues (16, 17). In dose fractionation, the total dose to be delivered is divided over several treatments, allowing normal tissues to recover between doses. RT can be hypofractionated, with a larger dose delivered over fewer sessions, or hyperfractionated, with many smaller doses received as often as twice a day. Conformal radiation, which is often used in conjunction with dose fractionation, involves using multiple beams that converge on the target tissue to deliver a higher dose there while reducing the dose to collateral tissues which are only in a single beam’s path.

In contrast to photon RT, CPT deposits a high dose of radiation as particles slow down within tissues, a phenomenon called the Bragg peak (Figure 1B). Because CPT releases more energy as ions slow down, the dose of radiation delivered to superficial tissues is much smaller than the dose delivered to deeper tissues. The depth at which the Bragg peak occurs can be varied by using particles of different energies. Since a single peak is often too small to irradiate the entire volume of a tumor, multiple beams of varying energies are used to overlap the Bragg peaks and distribute the dose to the entire tumor. The summation of these beams creates a spread-out Bragg peak (SOBP) (Figure 1C). By taking advantage of the SOPB, CPT delivers a high radiation dose to the targeted tumor tissue with minimal dose deposition to surrounding tissues. While the principals for delivering CPT is similar across types of ions used, there are differences in the dose deposition of different charged particles. For example, carbon-13 (^13^C), the most common experimental high-LET RT source, has a higher ratio of dose delivered in the Bragg peak to dose delivered in the initial plateau region (Figure 1B) compared to protons. The increased Bragg peak-to-plateau energy deposition of carbon ions results in higher energy deposition in target tissues with less collateral tissue damage and is the basis for the increasing clinical use of carbon ions.

Regardless of the type of radiation used, the first events after exposure that lead to cytotoxicity in healthy and targeted tissues are the formation of DNA breaks (18) and reactive oxygen species (ROS) (19) in tissues in the radiation path. In the nucleus, ionization leads to DNA breaks which in turn leads to aberrant DNA base pairs (20) and epigenetic changes (21, 22). In response to radiation-induced DNA breaks, multiple repair mechanisms are activated, most notably the ataxia-telangiectasia mutated kinase (ATM) and ATM- and Rad3-related kinase (23). These cause a signaling cascade that induces cell cycle arrest and DNA repair proteins *via* p53 (24). The complexity of DNA damage determines whether the cell will survive or whether apoptosis is initiated.

**Figure 2:**
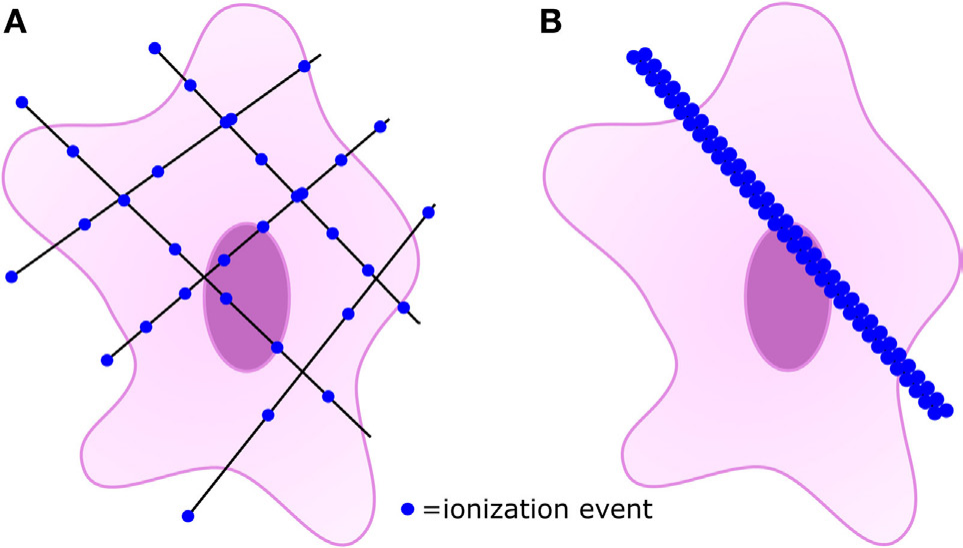
The main factor differentiating low-linear energy transfer (LET) and high-LET therapies is the amount of ionization along the path of the beam. **(A)** Low-LET radiation is sparely ionizing, and multiple exposures may be used to adequately irradiation target tissues. **(B)** High-LET radiation causes dense ionization along the course of the beam.

Low-LET photon beams cause diffuse and homogenous ionization and cause ROS formation throughout cells (Figure 2A), which mainly causes single-stranded DNA breaks (SSB). The sparsely ionizing low-LET therapies are most effective in the G2/M checkpoint of the cell cycle (25). In contrast, the high-LET beams of heavier ions cause dense ionization (Figure 2B) especially at the Bragg peak, and ROS rapidly associate with surrounding structures (26, 27). High-LET radiation causes more double-stranded DNA breaks (DSB) than low-LET RT (28). The resulting DSB are more complex and more likely to lead to cell death, whereas SSB are more easily repaired and are more likely to be sublethal (29). High-LET therapies are effective in all stages of the cell cycle, especially S-phase (27). Additionally, in experimental models, high-LET RT has shown the ability to overcome two major causes of tumor radioresistance: tumor stem cells (30) and hypoxia effect (31). Tumor stem cells are a subset of cancer cells believed to be a source of radioresistance because of increased antioxidant and DNA-repair capabilities (32). The tumor hypoxia effect refers to the decreased efficacy of radiation in hypoxic tumors. Although the exact mechanism by which tumor hypoxia leads to radioresistance is unknown, one hypothesis is that the presence of oxygen is necessary to create an organic peroxide intermediate with the broken strand of DNA induced by radiation. The peroxide then reacts with surrounding structures and fixes the break in place, contributing to signals favoring apoptosis. In hypoxic tissues, SSB are more likely to be reduced by surrounding sulfhydryl groups. The reduced carbon is more easily repaired and signals favoring repair and survival are induced. The DSB caused by high-LET radiation are not affected by oxygen concentration and mediate effective killing under hypoxic and normoxic conditions (2).

Most studies of radiation for oncological applications have focused on the effects of radiation in the nucleus, but more recently effects in the cytoplasm have become apparent and may be equally important to the study of RICVD. In the cytoplasm, ROS formation causes damage to the cell membrane, organelles (33), and the ligand-independent activation of multiple pathways, especially receptor tyrosine kinases (34). Not only does radiation directly produce ROS, it also causes ROS release from mitochondria (35). ROS disruption of normal cytoplasmic function and membrane structures can lead to cell death independently of or in conjunction with effects in the nucleus. However, in sublethally irradiated cells of collateral tissues, both nuclear and cytoplasmic damage results in signaling cascades that converge at signaling through nuclear factor kappa B (NF-κB) (36–38), which leads to multiple signaling cascades that suppress apoptosis, induce radioresistance, and induce inflammation (39).

## Mechanisms of RICVD

Reactive oxygen species formation has been shown to be an important factor in the development of RICVD, and decreased ability to clear free radicals causes a worsening of cardiovascular effects (19). ROS formation in healthy endothelial cells and subsequent signaling *via* NF-κB leads to an inflammatory state *via* expression of interleukin-1, interleukin-6, tumor necrosis factor-α (40), intercellular adhesion molecule-1 (41), and matrix metalloproteinases (42, 43). ROS levels remain elevated long after exposure to radiation. In animal models whose hearts were directly exposed to high-LET radiation, inflammation and apoptosis were shown to persist for at least 6 months (43, 44). This prolonged inflammation leads to a persistent but ineffective healing and remodeling response (45) marked by chronic inflammation of macrophages and mononuclear cells (46). The chronic inflammatory response is necessary for remodeling of damaged tissues, but the low levels of inflammation seen early in RICVD may be ineffective to fully restore tissue structure and function (47). Further, angiogenesis is disturbed after exposure to radiation due to the decrease in vascular endothelial growth factor secretion (48) and decreased tubule formation (14). The continuous attempts at repair induce the physiologic formation of more ROS (47), which contributes to a smoldering continuous inflammatory state. The vasculature’s inability to appropriately remodel from the initial radiation injury is further worsened by a decrease of endothelium-dependent relaxation (49, 50) worsening the effect of turbulent blood flood, another important factor in atherosclerotic development (51). Later, intimal thickening and atherosclerosis occurs, especially at areas of disturbed flow (44). The atherosclerotic effects of radiation are seen in models of low-LET radiation (52–54) as well as high-LET radiation (44, 49, 50).

Additionally, high-LET radiation has been shown to upregulate connexin-43 in the cardiac myocytes of animal models (55–58). Connexin-43 is implicated in the development of atherosclerotic plaques (59), and downregulation of connexin-43 has been shown to reduce atherosclerosis formation in animal models (60, 61). The exact role of connexin-43 in the development of RICVD is still unclear, but it likely plays a role in communication between vascular cells and inflammatory cells (62).

**Figure 3:**
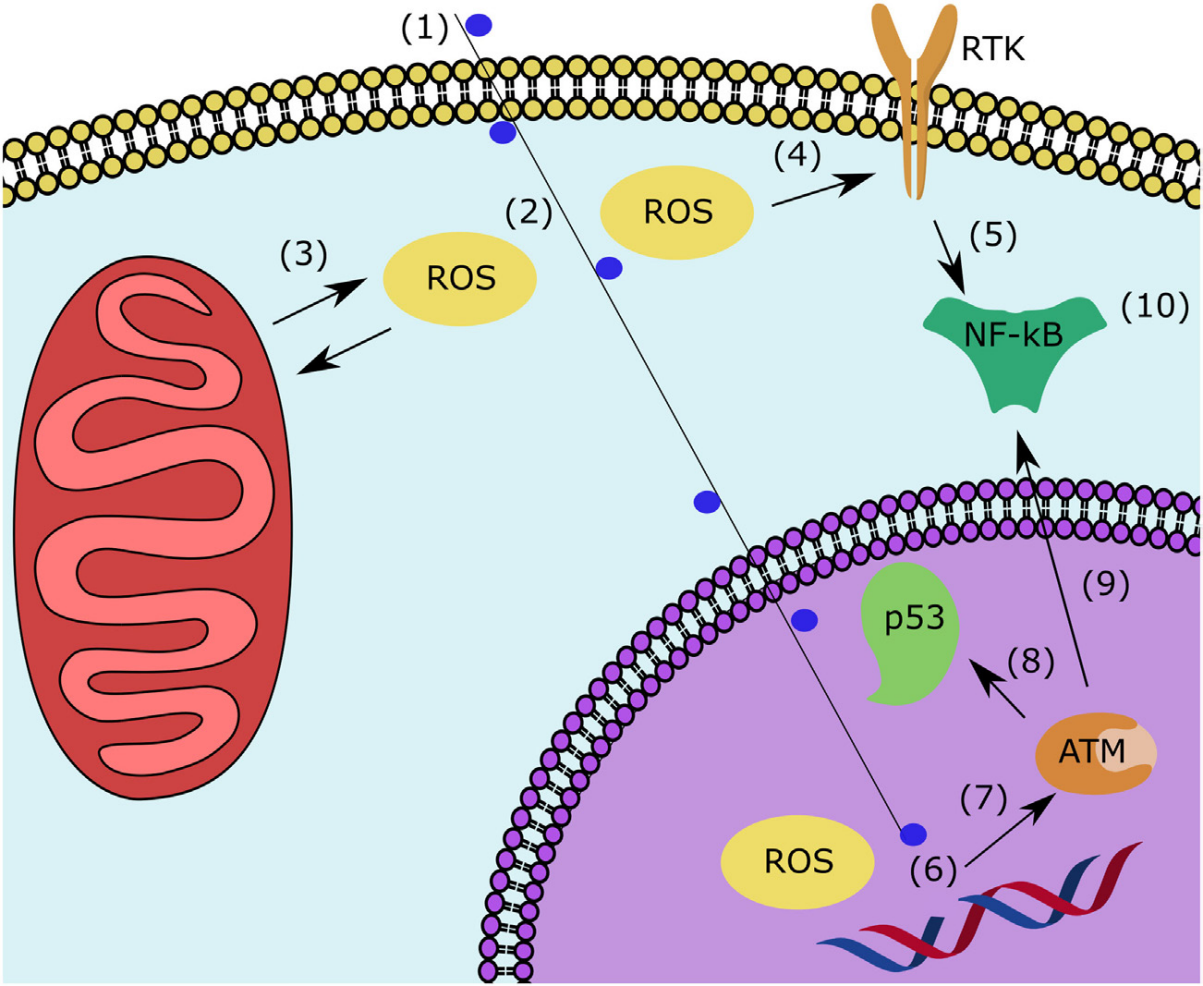
After sublethal irradiation, multiple events in the nucleus and cytoplasm contribute to the inflammatory state responsible for RICVD. In the cytoplasm, (1) sublethal irradiation of an off-target cell leads to (2) ROS formation and (3) ROS-induced-ROS release from the mitochondria. (4) ROS lead to ligand-independent activation of RTK which leads to expression of many pathways that (5) induce NF-κB. In the nucleus, (1) sublethal ionizing radiation leads to (6) ROS formation and single-stranded DNA breaks. (7) In response to DNA damage, ATM and ATM and AT and Rad3-related kinase are activated. (8) They induce p53 activation leading to cell cycle arrest and DNA repair as well as (9) inducing NF-κB. (10) NF-κB activation from both pathways induces factors that lead to antiapoptotic, radiation resistance, and inflammatory signaling. The presented mechanism is generally expected to occur in all sublethally irradiated cell types, but the pathogenesis of RICVD is first seen in the endothelial cells. Abbreviations: RICVD, radiation-induced cardiovascular disease; ROS, reactive oxygen species; RTK, receptor tyrosine kinase; NF-κB, nuclear factor kappa B; ATM, ataxia-telangiectasia mutated kinase.

Figure 3 illustrates the interrelation of proposed pathological mechanisms at the cellular level. In summary, sub-lethal DNA damage in the nucleus and ROS formation and release in the cytoplasm both activate NF-κB. NF-κB mediates a pro-survival and pro-inflammatory state in which ineffective remodeling leads to a vicious cycle of continuous ROS formation and persistent inflammation. The inflammatory state leads to impaired healing and endothelial dysfunction, making the vasculature more vulnerable to damage from non-laminar flow. Compensatory mechanisms manifest as intimal thickening and eventually atherosclerosis as inadequately healed endothelial injuries accumulate.

## Case Reports and Models of RICVD in High-Let Therapies

No large-scale clinical trial data is available for high-LET therapies. The first full trial with high-LET radiation began in 1994 (2), and most trials of high-LET therapy do not look at RICVD as an endpoint. Several small trials involving high-LET therapy near cardiovascular structures have been conducted, but none are large enough to determine the frequency or type of RICVD caused by these newer therapies. A case report showed the efficacy of carbon ion therapy against cardiac angiosarcoma, a tumor that is usually resistant to most forms of radiation and chemotherapy, without major off-target effects up to 1.5 years (63). In a case series published by Amino et al., eight patients treated for mediastinal cancer showed no cardiac toxicity up to five years post-^12^C irradiation. It should be noted that six of the eight patients were deceased at 5-year follow up due to non-CV-related progression of their disease (64). In small trials, carbon-ion therapy has been shown to be effective in controlling hepatocellular carcinoma, even inoperable tumors near the porta hepatis, and these trials have shown no acute effects to the vasculature of the liver (65–67). These preliminary results of the effectiveness of high-LET RT show promise for the treatment of tumors that were previously thought of as radioresistant, and the works do not show any evidence of acute RICVD. Increased sample size and longitudinal follow-up will be required to determine the rate of chronic RICVD caused by high-LET therapies.

Because of the paucity of clinical information about RICVD from high-LET therapies, most knowledge of the cardiovascular effects of high-LET radiation comes from animal and *in vitro* experiments. Mice are most commonly used to model the effects of RICVD. However, due to their inherently low plasma low-density lipoprotein (LDL) and high plasma high-density lipoprotein (HDL) coupled with a short lifespan, many murine strains are resistant to atherosclerosis, a principle measurable endpoint in RICVD. Mutant mice with defects in lipid metabolism such as ApoE- and Ldlr-knockouts and ApoE Leiden- and ApoB-100-mutants acting in a dominant negative fashion are often used in the study of non-radiation-related atherosclerosis (68). These models are also used for the study of RICVD because they increase the sensitivity of the mice to the cardiovascular effects and reduce the time to observable effects of atherosclerosis (69). The choice of mouse line used to model RICVD is important because different effects will be seen in mice of different genetic makeups (70).

Animal models used to study RICVD show similarities between the CV effects of high- and low-LET radiation. Loss of vascular reactivity appears to be a sentinel event and can be seen as early as 5 weeks postradiation in animal models (49, 50). High-LET causes an upregulation of genes related to cell senescence and oxidative stress (71), which is similar to the response seen after low-LET radiation. Further, supporting the role of oxidative stress after high-LET radiation exposure, the level of serum antioxidants are reduced after exposure, and antioxidant-rich diets reduce this effect (72). Xanthine oxidase contributes to ROS production and nitric oxide reduction in whole-body irradiated mice (19). The above animal studies use whole-body radiation to assess various aspects of the pathophysiology of RICVD. This approach is useful for eliciting broad response, but takes away from the improved targeting capability of CPT, i.e., the ability to avoid irradiating non-target tissues. Yu et al. examined the effects of high-LET radiation on vasculature using a targeted approach. In their study, non-irradiated arteries from test mice as well as arteries from sham-irradiated mice were used as controls for exposure to ^56^Fe radiation. They demonstrated that the high-LET ^56^Fe ions accelerate atherosclerosis in target arteries but not controls. They also demonstrated that different arteries have different sensitivities to high-LET IR in a similar manner to low-LET modalities (44). Additionally, studies investigating difference sequences of exposure to low- and high-LET radiation have shown differential cellular responses (73, 74). While animal models are useful for examining the phenotypic characteristics of RICVD, they have several setbacks including the expense required to maintain the mouse lines and time required for animal testing. Murine atherosclerotic models also have key differences from human atherosclerosis, such as location plaques occur, stability of the plaques that form, and structure of HDLs expressed (75), which may make it difficult to tease apart what may be subtle differences in the effects of low- and high-LET RICVD.

*In vitro* models are cheaper, faster, and offer more control than animal models. They allow for the isolation of parameters and simple measuring of outputs of cells *via* media and molecular techniques. Monocultures are most often used and may consist of cells derived from animal or human sources. Cell types commonly used to study CVD include human umbilical vein endothelial cells (76), cardiac myocytes, embryonic stem cells (77), and vascular support cells such as fibroblasts. High-LET ^56^Fe radiation has been shown to cause more DSB in HUVECS (14). The DNA damage caused by high-LET charged ions also appears more durable than those of low-LET radiation (78–80). Additionally, high-LET radiation more effectively induces endothelial cell adhesiveness which would contribute to inflammatory cell adhesion (41, 81). Reproducing CV cells exposed to high-LET radiation show sustained genomic damage and decreased functionality (77). The angiogenic capabilities of endothelial cells are more effectively reduced by high-LET radiation. It appears that the angiogenic inhibition is due to a decrease in secreted VEGF (48) leading to tubule inhibition in multiple endothelial cell types (14, 48). High-LET radiation has also been shown to reduce endothelial cell adhesiveness in culture which could be an analog for increased vascular permeability (42), and decreases the mitochondrial membrane potential due to leaking of ROS from the mitochondria into the cytoplasm (76). While most of the above studies have compared the effects of low- and high-LET radiation, LET is not the sole determinant of the cellular effects of radiation, and different types of radiation have been shown to have different effects even at the same LET (78). The *in vitro* techniques described above have provided useful insights into the mechanisms of RICVD such as DNA damage, cytokine response, and effects on individual cells. However, atherosclerotic development in RICVD is a multifaceted process likely occurring both acutely and over many years and involving multiple cell types. Traditional cell culture techniques may be insufficiently complex to appropriately model some aspects of RICVD such as matrix remodeling, reaction to disturbed flow, and cellular migration.

To address gaps present in animal and 2D *in vitro* models, 3-dimensional (3D) cultures are being considered for use as a model in studying RICVD (14, 78, 82, 83). 3D matrices can be used to construct multilayer cocultures that have a greater fidelity to the physiological state of tissues being test compared to 2D *in vitro* cultures. 3D cultures also offer more control, homogeneity, and ease than animal models. Hydrogel models can be used to examine cytokine and morphological changes in response to stimuli. Constructs of vascular endothelial and interstitial cells can be used to replicate endothelial cell behavior (14) and model the damage caused after radiation (82). Flow cells can be used to recreate shear stress from blood flow (84) including pathological shear on vessel walls. 3D cultures have been especially useful in studying the effects of low- and high-LET radiation affects microvasculature. In a series of studies, Grabham et al. showed that the damage caused by high-LET ^56^Fe ions on both mature and developing vessels compared to proton or photon IR which preferentially affected developing vessels (78). Additionally, they used 3D culture to show that ^56^Fe radiation inhibits late stage angiogenesis, namely endothelial cell migration and tube formation, rather than early motile tip and intracellular adhesion, which is inhibited by low-LET radiation (14). The development and testing of more complex 3D models is underway and may provide new insights into the pathogenesis of RICVD.

## Implications on Treatment

The link between radiation and CVD is well established in human cohorts at doses greater than 0.5 Gy (85). Interestingly, low-dose high-LET radiation may have some anti-inflammatory effects in a dyslipidemic murine model (86), but the dose-rate and state of the disease affect the modification radiation has on atherosclerosis progression (87). The dose cancer patients receive varies widely for the disease being treated but are often well in excess of the doses known to cause RICVD (9, 88–90). The importance of cardiovascular health during and after treatment is well recognized, but there is still a lack of national guidelines (91).

The prevention and treatment of RICVD consists of optimizing traditional cardiovascular risk factors including hypertension, blood sugar, heart failure, coagulability, and blood lipids, as well as encouraging healthy diet, exercise, and medication adherence. RICVD may be resistant to some aspects of treatment (92, 93), making the optimization of all modifiable factors important in patients undergoing treatment. Additionally, patients often receive surgery and chemotherapy with radiation for the treatment cancer, and certain chemotherapies, such as anthracyclines and trastuzumab, are known to be directly toxic to the heart. The presence of CVD is not an absolute contraindication to the use of RT. Rather, clinicians administering cardiotoxic drugs with or without radiation should keep in mind patients’ comorbidities and risk factors and weigh them against the therapeutic advantage granted in terms of tumor control (94).

## Relevance of RICVD to NASA

The effects of radiation are also of interest to NASA (95), as well as other space programs, as it poses a significant risk for manned spaceflight. RICVD is among the radiation-related health risks of concern. The types of radiation found in the space environment are significantly more damaging than those found on Earth and include galactic cosmic radiation (GCR), solar particle events, and trapped protons and electrons. GCR consists of high atomic number and high energy (HZE) nuclei, like carbon and iron, as well as high energy protons (96). There are similarities with charged ion RT which uses single ion beams of carbon or proton. Differences between the space radiation environment and clinical RT protocols includes dose levels, dose-rates, whole body vs. partial body irradiations, along with the mixed ion fields present in space versus single ion beams used for CPT.

National Aeronautics and Space Administration maintains a research portfolio to evaluate effects of high-LET radiation on CVD in order to characterize and mitigate radiation risks posed to astronauts on exploration missions (95, 97). Evidence comes from a body of cell and animal work as well as from terrestrial epidemiology analyses of atomic bomb survivors and nuclear workers showing a demonstrated risk for RICVD at doses greater than 0.5 Gy (6). However, at lower, space-relevant doses and radiation types, the association between exposure and cardiovascular pathology is more varied and unclear. Recent work has reiterated that, to date, there is no evidence in the astronaut cohort of increased risk of CVD (98, 99). This confirms the healthy worker effect expected in an astronaut population but also highlights the limitations of such a cohort, including small sample size and large confounding effects as well as the relatively low doses of radiation that astronauts have experienced to date. Exploration missions with longer durations and outside the LEO will result in larger radiation exposures to the astronauts, and a mission to Mars predicted to last several years (95, 100) will result in doses nearing the 0.5 Gy threshold for RICVD observed in terrestrial cohorts. Therefore, NASA requires risk characterization and mitigation strategies for the risk of RICVD for a Mars mission or other longer exploration missions. NASA relies on cellular models (both 2D and 3D), animal studies, and ongoing epidemiological analyses with both low- and high-LET exposures to inform its knowledge gaps. This research strategy is detailed within the NASA Human Research Roadmap (97), where current and planned work is described within the eight knowledge gaps for the “Risk of Cardiovascular Disease and Other Degenerative Tissue Effects From Radiation Exposure and Secondary Spaceflight Stressors.” Advances within NASA’s research program as well as within terrestrial work with CPT can inform both the risk of RICVD as well as mitigation strategies. Specifically, countermeasures already approved for use for CVD and evaluated in clinical radiotherapy cohorts will be a first priority for mitigation strategies in astronauts for RICVD.

## Conclusion

The increasing number of patients being treated with radiation justifies further research into mechanisms and druggable targets for treatment and prevention of RICVD. High-LET CPT is an innovative form of RT that shows promise in the early phases of testing. There is not sufficient clinical data to draw conclusions about the efficacy of high-LET RT, and cost remains a practical barrier to studying and implementing of CPT (101, 102). Still, its potential to overcome radioresistance in tumors and improve targeting around sensitive organs warrants further research.

In terms of RICVD, the complex pathophysiology remains to be fully elucidated. Population data does not yet show any increased risk of CVD in populations exposed to <0.5 Gy. *In vitro* models have shown that multiple variables beyond total dose contribute to differential responses. Factors shown to be important in cardiovascular cells’ response to radiation include dose rate, LET, particle type regardless of LET, and genetic makeup of the model being used. Better models, such as 3D cocultures, which are more representative than standard 2D cell culture and faster, cheaper, and more tunable than animal models, are currently under development for use in the study of RICVD. They may offer even better insight into pathological progression after exposure to radiation. Finally, while most studies of RICVD revolve around cancer patients, this information is also relevant to NASA. Future space missions will be longer and outside of the earth’s magnetic field, exposing astronauts to greater radiation doses. NASA’s current research on RICVD, which relies on cellular and animal ground-based studies, can both contribute to and benefit from concurrent work informing radiotoxicities resulting from cancer therapy.

## Author Contributions

CS wrote the manuscript and prepared the figures. J-iA, ZP, and KG-A contributed to conception and editing of the manuscript.

## Conflict of Interest Statement

ZP is employed by KBRwyle. All other authors declare no conflict of interest.
